# Medical students’ awareness of overdiagnosis and implications for preventing overdiagnosis

**DOI:** 10.1186/s12909-024-05219-2

**Published:** 2024-03-08

**Authors:** Lucinda Colbert, Iman Hegazi, Kath Peters, Natalie Edmiston

**Affiliations:** 1 Hervey Bay Hospital, Wide Bay Hospital and Health Service, Hervey Bay, QLD Australia; 2 School of Medicine, Western Sydney University, Campbelltown, NSW Australia; 3 School of Nursing and Midwifery, Western Sydney University, Campbelltown, NSW Australia; 4 University Centre for Rural Health, Lismore, NSW Australia

**Keywords:** Medical education, Medical student, Overdiagnosis, Diagnosis, Clinical placements

## Abstract

Overdiagnosis is a growing health issue, yet our understanding of medical students’ exposure to this concept within medical education is limited. Our aim was to explore students’ experience of diagnostic learning to identify how overdiagnosis may be understood by students. During in-person and online semi-structured interviews throughout 2021, we explored the education experience of twelve Western Sydney University medical students in years 3–5. Through inductive thematic analysis we identified four themes. These themes encompassed student commitment to learning about diagnosis, lack of certainty surrounding diagnosis and emotional factors of medical care, overdiagnosis as seen through the lens of high and low-value care during clinical placements and student-identified missed learning opportunities related to overdiagnosis. This study found that medical students develop inherent knowledge of overdiagnosis through an interplay of personal factors, medical school curriculum and the setting in which their training takes place. Our findings allow insight for future improvement of medical curriculum to produce exceptional medical graduates.

**Supplementary Information:**

The online version contains supplementary material available at 10.1186/s12909-024-05219-2.

## Introduction

Overdiagnosis is a complex concept that broadly means making people “patients” unnecessarily. It includes overdetection of problems that were never going to cause harm and overdefinition or expansion of disease definitions to include either normal human experience or risk factors as disease [1, 2]. Overdiagnosis interlinks with overinvestigation and overtreatment under the banner of ‘too much medicine’ and is a key feature of low-value patient care [1]. The possible outcomes of overdiagnosis are significant, encompassing psychological effects of disease labelling, physical harm due to unnecessary interventions, reduced quality of life, and wasted resources [1, 3, 4]. Several drivers contribute to overdiagnosis in the clinical setting including medical culture, the healthcare system, healthcare professionals, patients and the public [5]. Health professionals are likely a key driver of overdiagnosis [5, 6]. Doctors in particular are responsible for formally making a diagnosis and developing an associated treatment plan and hence have a key role in overdiagnosis prevention [1, 5]. One strategy for avoiding overdiagnosis is considering quaternary prevention, the actions that can be taken to protect individuals from medical interventions that are likely to do more harm than good, in each patient context [7]. Thinking how to best protect patients from potentially harmful interventions should be routine in patient work up to avoid low-value care and overdiagnosis [7]. Current literature suggests targeted education for medical students is critical to preventing overdiagnosis and low-value care in clinical practice [8–11].

Many Australian medical schools support students through a vertically integrated curriculum [12]. This framework fosters continual learning and patient-centred care by providing foundational knowledge through early patient contact, problem-based learning, and evidence-based medicine [13, 14]. Early clinical learning forms the foundation of diagnostic performance [12] and integration of learning principles allows a curriculum structure that supports and enhances education [15]. Evidence suggests repeated exposure, self-reflection, feedback and refinement of concepts are crucial for consolidation of learning [15]. Medical school is an opportunity for students to cycle repeatedly through these learning stages and build the foundation for clinical practice and future learning.

Within the early years of the medical program, the refinement and feedback stages may be in the form of tutorials with peer and teacher discussions. In the later years, students are typically shadowing and working within medical teams during full-time hospital and general practice attachments. These clinical placements provide immersive learning opportunities where students begin to focus on clinical reasoning and gain some responsibility for patient care as they progress throughout their degree [16].

During clinical placements, feedback comes from colleagues, peers, and patients and is highly dependent on the placement which may differ in rurality, specialty and team structure. Whilst self-directed learning is a valuable tool, particularly important for medical students and professionals, feedback is integral to the development of critical thinking and clinical reasoning [15]. Critical thinking involves cognitive processes used to analyse knowledge and is not situation dependent [17, 18]. In contrast, clinical reasoning is the cognitive and metacognitive process used to analyse knowledge within a clinical context or for a specific patient [17, 18]. It is a complex process that includes gathering, prioritising, synthesising, and analysing data to formulate a clinical hypothesis or conclusion [17, 18]. Clinical reasoning heavily relies on critical thinking skills, and these two cognitive processes are intricately intertwined [17–19]. Medical schools have a role in developing students’ clinical reasoning to be inclusive of the concept of overdiagnosis and the potential harms of overdiagnosis.

There is little in the published literature that explores the student perspective on overdiagnosis. Our research team identified this gap and aimed to explore medical students’ perspectives and how aspects of medical education interplay to form students’ foundational knowledge related to overdiagnosis.

## Methods

### Aim

The aim of this project was to determine medical students’ awareness of the concept of overdiagnosis by exploring their learning of diagnosis and clinical reasoning through a lens of high and low-value care.

### Setting

The study occurred at Western Sydney University (WSU); an undergraduate medical school located in New South Wales, Australia. The medical school program encompasses two on-campus learning years and three full-time clinical rotation years. Early-stage learning includes lectures, tutorials, and an introduction to the clinical learning program with small-group tutorials at partnering teaching hospitals. In their later years, students rotate through core medicine and surgery rotations and complete a specialty medicine rotation year allowing full immersion into the clinical environment. The university awarded a Bachelor of Medicine, Bachelor of Surgery (MBBS) to graduates until 2022 and will award a Doctor of Medicine (MD) to graduates from 2023. The study took place in person and over zoom and included twelve WSU students.

### Recruitment and data collection

We developed a semi-structured interview protocol (Supplementary File 1) that explored preclinical and clinical education to allow student reflection and identification of inherent knowledge and learning of clinical reasoning related to diagnosis. Questions encouraged students to describe their preclinical and clinical learning of ‘diagnosis’ and how they approach patient workup. Our interview protocol included generalised questions relating to medical education and more targeted questions to highlight student experience and understanding of high and low-value care. Considering the range of experience and knowledge of the student group, we utilised high and low-value care as a mechanism to identify students’ inherent knowledge of overdiagnosis. We recruited students through poster advertisements, social media, and email announcements. Eligibility criteria included students in years 3–5 who had not taken part in a study of a similar topic. In-depth semi-structured interviews were conducted both in person and over zoom from May-August 2021. Interviews were audio recorded, transcribed, deidentified and tagged with a number combination.

### Data analysis

Data were analysed inductively, following the Braun and Clarke phases of thematic analysis [20]. After familiarisation by thorough perusal of the data, codes were generated across the entire data set. Codes were revised and the transcripts were reviewed and coded a total of three times to produce the final data set in August 2022. With each coding round there was progression from descriptive codes to latent codes.

The coded data were collated and analysed to identify overarching themes.

Codes and themes were discussed extensively by members of the research team to ensure they represented the data set. Further analysis was conducted to define the themes and select examples to construct the report [20]. A framework was developed through collaborative discussion outlining how curriculum and education influenced student perspectives and knowledge of overdiagnosis and ability to avoid overdiagnosis.

### Rigour

The interview protocol was reviewed and tested with the members of the research team prior to formal interviews taking place. The first author conducted all the interviews utilising open-ended questioning and transcribed the audio for continuity. All coding and inductive thematic analysis were performed by the first author then discussed and critically reviewed by the research team.

Data collection concluded once interviews yielded no new themes indicating that thematic saturation was achieved. The data set included twelve interviews from students of a diverse background accounting for age, gender, location, cultural background, and year level.

The first author was a medical student and peer of participants. This minimised potential influence by senior investigators and ensured credibility of the research findings. Having a peer researcher also allowed a unique perspective in the development of the interview protocol and an interview approach that was non-judgemental, and without significant power discrepancies. As an Aboriginal person herself, the first author was able to reach students of Aboriginal and Torres Strait Islander descent during recruitment and create a safe environment for study participation. The other research team members included: two medical doctors, one the Director of Medical Education, the other a researcher in medical education, and an experienced qualitative researcher. The different perspectives of the researchers and frequent opportunities for reflection, both individually and collectively, increased the credibility and dependability of the research.

### Ethics

The literature review, outline of the project, interview protocol and recruitment documents were submitted, and ethics approval was granted by the Human Research Ethics Committee at WSU under the project title ‘*Too much medicine’ in the medical curriculum*. Approval was as an amendment to ‘Connecting Up: A program of research to evaluate the WSU Medical Program’; ethics approval number H9989. Prior to the commencement of interviews, participants were informed of the study details and aims via an electronic participant information sheet. Written informed consent was obtained, with verbal informed consent reiterated at the commencement of each interview.

## Results

Twelve students from years 3–5 at WSU participated in the study. Participants had diverse cultural backgrounds with students identifying as Australian, South Asian, Pacific Islander, South American/European and four as Aboriginal and/or Torres Strait Islander. Participants’ ages ranged from 21 to 27 years. Four participants indicated they were a rural student, having lived rurally and/or completed long-stay rural education in healthcare (Table 1).

**Table 1:** Participant demographics

	Year Level	Identified Gender	Rural student
**Participant 1 (P1)**	Four	Male	No
**Participant 2 (P2)**	Four	Female	No
**Participant 3 (P3)**	Four	Male	No
**Participant 4 (P4)**	Three	Male	No
**Participant 5 (P5)**	Three	Female	No
**Participant 6 (P6)**	Three	Male	No
**Participant 7 (P7)**	Five	Male	Yes
**Participant 8 (P8)**	Five	Female	Yes
**Participant 9 (P9)**	Three	Female	Yes
**Participant 10 (P10)**	Four	Male	Yes
**Participant 11 (P11)**	Three	Female	No
**Participant 12 (P12)**	Five	Male	No

Four themes emerged regarding the student experience of medical education and the relationship to learning about overdiagnosis:

- Students high level of commitment to learning about diagnosis meant further learning occurred independently or with peers.
- Students lacked certainty regarding diagnosis; in earlier years this was experienced as frustration but in later years reflection and clinical experience allowed uncertainty to be understood as inherent to clinical practice.
- High and low-value care as a lens for learning overdiagnosis and the role of clinical placements in developing students’ clinical reasoning skills.
- Missed learning opportunities and issues in medical education related to overdiagnosis identified by students.

### Students high level of commitment to learning about diagnosis meant further learning occurred independently or with peers

Students demonstrated high-level engagement with learning material and external resources in and around prescribed university hours. Participants explained how their extracurricular activities tied into their learning, including listening to podcasts targeted towards Australian junior doctors, through to reading journals and research publications to expand their knowledge. In addition to required tutorial attendance, participants recalled visiting hospital wards in small groups to practice clinical skills with patients and receive peer feedback. Students described connections between previous employment in the medical field and their placement learning and how they can now identify and understand low-value care practices in the clinical environment.

Students identified after-hours, peer-led tutorials as important in furthering their knowledge and clinical reasoning skills in the context of patient case examples and demonstrated commitment to their education by identifying learning opportunities for themselves where patient interaction was reduced during the COVID-19 pandemic.

“The best teaching that I have had has come from working collaboratively with other students…they were really, really good about going through things that we talked about in the day.” (P9)

While students described these additional learning opportunities to be beneficial to the development of their clinical reasoning, not all students were engaged with the same additional learning opportunities resulting in a disparity in experience. Students also appreciated that theoretical learning has its own limitations and patient case exemplars often have significant leeway for development of ideas that does not necessarily represent real patient situations. Patient case scenarios were noted to be useful for diagnostic learning however students appear to be removed from concepts of investigation availability, overdetection and cost.

### Students lacked certainty regarding diagnosis; in earlier years this was experienced as frustration but in later years reflection and clinical experience allowed a degree of uncertainty to be understood as inherent to clinical practice

When asked how to approach a patient in the clinical setting students answered unanimously that initial workup included eliciting a comprehensive history and performing a targeted examination before considering investigations and seeking guidance from clinicians. Students expressed confidence in practicing the foundations of patient-centred care. They could identify the need to think critically and use information from discussions with the patient, general inspection of the patient and environment, the patient’s body language and information from the history and examination to make clinical decisions and develop a diagnosis. With a strong foundation, the initial workup would inform investigations and reduce the instances of low-value care and incidental findings.

Students became unsure of themselves after the initial workup. They expressed worry that they lacked experience to make complex diagnoses and were compelled to order extensive and invasive investigations in clinically ambiguous scenarios. Emotions including frustration when they could not find an answer and fear they missed something that would cause adverse patient outcomes emerged in student interviews. These negative emotions were identified as contributors for broadening differential lists and resorting to investigations to guide their clinical reasoning rather than utilising clinical reasoning skills to inform work up.

“What if the thing that you think is unlikely is actually the answer? And what if that is life threatening, then what do you do…?” (P9)

Students felt medical education is geared towards diagnosis. They were uncertain how to proceed without one and how to manage patient expectations of them as a medical professional. Classes designed to form the foundations for patient workup have been identified as a precipitate for uncertainty as students struggle to balance common patient presentations and broad thinking in clinical decision making, becoming conflicted and nervous about making premature decisions in case they are incorrect.

“I find it…frustrating because I guess that’s what my education has been geared towards. It [diagnosis] shouldn’t be a necessary part. But it feels like a necessary part” (P6)

Analysis of responses from early clinical students in comparison to those of final and penultimate year students emphasised the advancement of critical thinking skills when students are supported in developing their clinical reasoning skills. There was evidence that upon reflection, students could understand that in some instances there will be no clear-cut diagnosis and that high-value medical care can still be provided. They recognised that they didn’t need to have answers to everything, and that support was available in the workplace. As students progressed in their medical careers it was clear that they were becoming comfortable with a degree of uncertainty and recognised this as normal in medicine. The distinction between uncertainty due to lack of knowledge and clinical reasoning skills and uncertainty as a normal part of the diagnostic process informed by clinical judgement was obvious between early clinical students and final year students particularly. Many students also appreciated that frustration and fear should not be motivators for further investigation and that patient-centred care takes priority.

“When I was younger, I felt a little bit more helpless and a little bit more frustrated…that I didn’t have answers. Now… my priorities have shifted…it’s not always necessarily about finding an answer to a condition, rather trying to help the patient achieve their goals.” (P1)

### High and low-value care as a lens for learning overdiagnosis and the role of clinical placements in developing students’ clinical reasoning skills

During clinical placements, students observe how doctors interact with patients and learn from their justification of clinical decisions. Direct teaching also occurs in the clinical environment. Increased exposure and opportunity for students to ask questions enables the distinction between high and low-value care practices to emerge.

Although students observe low-value care practices within the hospital, not all students perceive it as low-value care. Students presume their supervisors are practicing and teaching guideline informed high-value patient-centred care and subsequently model their own clinical practice from what they are witnessing on placement. In fact, evidence from most interviews suggests that often students are witnessing low-value care inclusive of overinvestigation and overdetection. These students then approaching patient workup without a strong clinical reasoning skillset, are quick to consider extensive investigations before appropriate consideration of the risk benefit profile and are not sufficiently learning about overdiagnosis to avoid it.

Some students described teaching scenarios where supervisors justified their clinical decision making and discussed how clinical findings contributed to diagnosis and treatment plans allowing them to understand the concept of high-value care. General practice, critical care and rural placements were identified as high impact learning opportunities due to variety of presentations and increased opportunities for students to see patients independently. Students who attended placement in rural hospitals described the supervising team encouraging them to workup patients independently and engaging them in weekly long and short cases with scheduled time for discussion. This facilitated direct feedback and increased student’s clinical confidence and competence.

“In (rural location)…I was given a lot of opportunity to see patients on my own. And instead of just… where you see it with the team, and…not really do a whole lot, your registrar would send you…to do a short case on a patient, take history, take an exam, come back…and tell me what you think.” (P8)

Rural students consistently identified high-value care practices and described witnessing their supervisors utilise history and examination, to inform clinical reasoning and investigations, more than metropolitan students. Considering patient’s values, information from the workup, and patient expectations enabled sound clinical judgement and conservative management.

“[Supervisors] all take a history and an exam, even if the patient has…been admitted… If they haven’t seen the patient, they’ll still take their own history and do their own examination…you need to see it with your own eyes, and you need to make your own interpretation of what the patient’s presenting with.” (P8)

“I think that overdiagnosis goes hand in hand with overinvestigation…you need to know when it’s clinically appropriate.” (P8)

As such, rural students could describe overdiagnosis and low-value care drivers more clearly than others, having had more exposure and experience practising high-value clinical workup in areas that do not have the same resources or accessibility as metropolitan hospitals.

In large Australian hospitals, where most students conduct their placements, resources are readily accessible, and students perceive investigations as an easy fallback. Considering routine investigations performed every day, some students had difficulty distinguishing reliance on investigations and clinically indicated investigations and could not understand how so-called routine investigations could cause patient harm.

“If they [patients] come in with something obvious, you’re going to do a basic CT, X-ray to find out what’s wrong.” (P5)

Students recalled that patient distress is also a factor that drives medical professionals to continue investigative workup even with minimal clinical indication. When patients are desperate for an answer or are receiving care below their expectations, patients’ frustration can be transferred to the treating team, increasing pressure for the team to provide clearcut answers.

“A lot of the frustration comes from a patient, and it lands on the doctor…I think that puts a lot of pressure on the medical teams to keep searching and finding things.” (P9)

With increased investigation, students recalled incidental diagnoses. Whilst many students had not heard of overdiagnosis in their medical careers, it was obvious they had begun to learn about the concept inherently. Students could identify the relationship between overinvestigation and the discovery of incidental, noncritical diagnoses. They could identify patients receiving treatment for incidental diagnoses or multiple treatments for the same condition that were of no benefit. Many students could explain the dangers of screening, particularly prostate specific antigen screening and understood the controversy surrounding these tests. However, it was evident in most interviews that there were situations where reliance on investigations and low-value care practices were not identified. For some students this was more noticeable than others and may be tied to their level of experience. Lack of awareness of low-value care and the drivers of ‘too much medicine’ can mask these practices to students observing them in the clinical environment and can lead students to adopt these thought patterns and practice styles.

“Nowadays…they’re [investigations] essential… you can’t make a diagnosis without them” (P10)

### Missed learning opportunities and issues in medical education related to overdiagnosis identified by students

Students identified missed opportunities for learning about clinical reasoning. For problem-based learning (PBL) classes, patient cases were allocated to specific teaching blocks which allowed students to formulate diagnoses based on the current learning topic. Participants recalled PBL experiences of students calling out as many diagnoses and investigations as possible, rather than suggesting answers found from clinical reasoning. Although students commented that PBL was their initial preclinical exposure to diagnosis and formed their foundational understanding, they also suggested the lack of clinical reasoning in PBL impacted their ability to narrow down differentials in clinical years.

“In PBL, we tried to do a lot of diagnosis. In preclinical years diagnosis is painted as very black and white. Whereas in reality, it’s never the case… in clinical practice, you often don’t reach a diagnosis, you simply manage and move on…. I know in PBL, we try to come up with lots of different things. But… I often think it’s not a good way of doing it, because we’re not encouraged to consider the whole picture when we’re shouting out diagnoses… But often, it’s presented in a way that’s very clear cut, which is helpful for learning the process of diagnosis, but I guess it’s not reflective of reality.” (P6)

Students frequently commented that they were not directed to consider clinical justification or implications of diagnoses and investigations that they were suggesting. Comments arose that it did not matter how they communicated in the case since the information was going to come on the next slide regardless of their clinical reasoning or patient rapport. Not only was PBL described as not reflective of clinical practice, but students suggested that these classes hindered their decision making as they progressed through the years. Students described the differences in teaching styles and tutor engagement in PBL with some tutors focussing on diagnosis or clinical reasoning while others left students to their own devices and did not engage with the class or redirect students to formulate their clinical reasoning skills.

Students reported that without the confidence that should have been gained from these classes they were more inclined to consider extensive investigations to narrow down a broad differential list to compensate for lack of clinical reasoning. Further, they described reluctance to commit to common diagnoses despite a supporting clinical presentation leaving them more susceptible to overinvestigation and overdetection.

“In my experiences, we threw out as many diagnoses as we could think, and then…as many tests as we could think…Without often thinking about what was or wasn’t really needed.” (P2)

Teaching relating to clinical examinations followed a strict proforma that required adherence to pass final exams, however students commented that this proforma is not indicative of medical practice. They also identified lack of understanding beyond the proforma of what abnormal findings represented especially since these exams were mostly performed on healthy individuals with no abnormal examination features. Students found that there were no explicit opportunities within the clinical medical curriculum to practise and receive feedback on their individual clinical reasoning which left them to find these opportunities as best they could on their own thus delaying their clinical development.

“I could recite a gastro or a cardio…exam off the top of my head, like it’s a script from some theatre performance…that isn’t necessarily applicable to real practice…the focus point of a lot of it is pass the exams, not learn to be a good doctor.” (P9)

In the clinical environment, students identified lack of time and heavy patient loads as factors impacting education. Differences in supervisor values and experience affected the standard of teaching, with some supervisors choosing not to explicitly teach and some providing teaching at an inappropriate level. Participants with supervisors who scheduled regular teaching or incorporated learning opportunities into ward rounds with realistic patient examples described better understanding of clinical justification and reasoning techniques. Without the support of this teaching during clinical years students felt overwhelmed by complex clinical scenarios and would be inclined to regress back to relying on investigations to form the foundation of their workup.

## Discussion

This study has found that medical students develop inherent knowledge of overdiagnosis through an interplay between personal factors, the medical school curriculum, and the clinical setting in which their training takes place.

**Figure 1.**
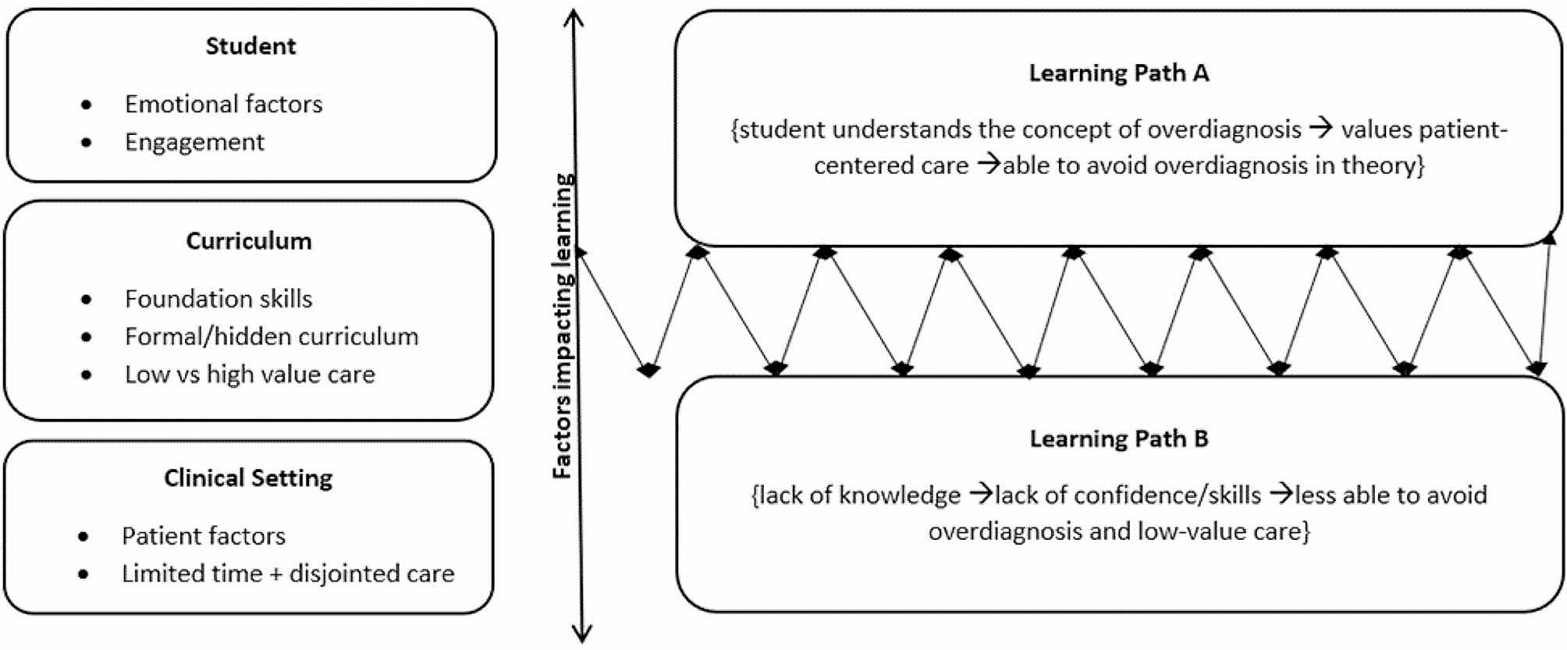
Model of student experience in learning. This model was developed from the results of our study to highlight factors impacting the learning pathway of students. Since learning is dynamic, students can move between learning path A and learning path B depending on their experiences and progression. From our results there are three key influences on education: the student themselves, the curriculum, and the clinical environment the student is engaging with. **Learning Path A** Describes a student that has a thorough grasp of clinical reasoning and understand the rationale behind investigations, treatment, and management. This student can practice aspects of patient-centred care in their placements and consider how to avoid low-value care. The student can describe the concept of overdiagnosis and identify it in the clinical environment; this may not mean the student knows the term overdiagnosis. This student can identify the impacts of emotion and when to ask for help. They have been taught principles of high value care and have witnessed these role-modelled by clinicians. Poor experiences and lack of consistent teaching can push students down to learning path B. **Learning Path B** Describes a student that may have limited knowledge or experience, who is not confident in themselves or their clinical reasoning. These students are unaware of the concept of overdiagnosis and may not be able to identify low-value care practices. As a result, students in learning path B cannot intentionally avoid overdiagnosis and low-value care. Limited time and disjointed teaching on placements contribute to deficits in students’ learning. Formal teaching and role-modelling of high-value care and opportunities for students to practice clinical skills and seek feedback can allow students to progress and move up to learning path A

Our results indicate two learning pathways whereby students are aware of and can actively take steps to avoid overdiagnosis, or through lack of experience or knowledge, students are more inclined to inadvertently engage in overdiagnosis. Students move between learning path A and B based on their clinical experiences and education. We hypothesised a model (Fig. 1) explaining the relationship between the student, curriculum, and clinical setting and how students’ learning paths are influenced.

Already known to be a driver of overdiagnosis, we anticipated students would talk about their experience with uncertainty in clinical medicine and aimed to explore their perspectives [5, 8, 21]. Our results mirrored studies exploring clinician perspectives, however, we were able to identify contributing factors at the student level [9]. Students described lack of confidence based on their experience levels, fear of harming the patient by missing clinical clues, and inability to narrow down differentials due to thoughts of ‘what if?’ stemming from their early year experiences. Our participants and wider medical education literature acknowledge the values of problem-based education in supporting the framework for clinical decision making and diagnosis [13, 22, 23]. PBL is designed to encourage critical thinking and introduce students to clinical cases, diagnostic workup, and communication skills [23]. However, without tutor direction and feedback this education format loses effectiveness [23]. A 2014 paper exploring overdiagnosis in paediatrics described a “shotgun approach” to diagnosis evident in PBL where the environment fostered possibilistic and unusual diagnoses as opposed to probabilistic differentials [8]. Our students frequently commented on their experience in PBL encouraging wide-ranging and unusual diagnoses with little thought to diagnostic accuracy or feasibility based on patient presentation. Encouraging thoroughness without also teaching clinical reasoning skills contribute to overutilisation and overdiagnosis [24]. Students recalled that it wasn’t until their clinical placement that they realised this approach to differentials was to their detriment, describing reluctance and fear of committing to clinically-sound differentials. While the foundation of PBL is student-led learning and discussion, tutors play a crucial role in maintaining an effective group dynamic that fosters cohesive collaboration and critical thinking [22, 23]. Tutors need to be able to guide students to formulate learning objectives while working stepwise through clinical cases [22, 23]. Clear facilitation, feedback, and redirection when a group veers off track assists in preventing this “shotgun approach” [22]. It became evident that issues in PBL were not confined to one institution and considering overdiagnosis, overutilisation and patient harm as just a few consequences of impaired clinical reasoning, it is recommended that clinical reasoning feature more prominently in early medical curriculum [25].

The culture of medicine is known to encourage constant action, with benefits of investigations being the forefront of teaching rather than potential patient harms [8, 11]. When supervisors practice low-value care, medical students absorb these practices and develop ambivalence towards value [26, 27]. Reliance on investigations contributes to overdiagnosis and can go unnoticed in the absence of a curriculum focussed on clinical reasoning. WSU operates a vertically integrated curriculum which has proven to be beneficial in supporting students in practicing high-value care [12, 14]. Studies have shown that in addition to the inherent skills fostered within vertically integrated curriculums, students can identify some low-value care practices in the clinical setting [26]. Participants in this study themselves could identify low-value care and overdiagnosis. Concerningly, however, several students considered this routine practice. This demonstrates that vertically integrated curriculum alone is not sufficient to instil high-value care practices, and that supervisors require specific training to contribute to student perceptions of best-practice care.

Students in the rural program achieved increased clinical competence and were more readily able to describe high-value care. These students demonstrated application of strong clinical reasoning that will assist them in avoiding overdiagnosis in the future. Students who train rurally not only anecdotally describe better learning experiences but are known to have better access to mentorships, small group learning and independent patient care opportunities [28, 29]. The diverse patient populations in rural health settings contribute to a varied education and with less accessible resources in comparison to metropolitan centres, the emphasis is placed back on clinical reasoning skills [29]. Students in high-healthcare intensity regions have been shown to have decreased exposure to high-value care during their training which may be contributing to overdiagnosis since clinical reasoning skills are not as well developed [26]. Students as a collective from the literature, and our own study, report insufficient teaching and desire for change in medical education [26, 30]. Whilst students may inherently understand overdiagnosis, this is clearly not sufficient to instil clinical confidence or avoid overdiagnosis.

### Implications

The findings of this study suggest that students’ perceptions of patient care and overdiagnosis are influenced by their education and experience in the clinical setting, and that their learning pathways are dynamic and evolving. Evidence revealed that some students can appreciate and practice high-value care and may be able to support students who are not yet at this level. Further experience and increased opportunities for practical application of skills can enhance clinical confidence at the student level. This could be supported by measures to improve the teaching skills of supervisors to enable high impact education on clinical placements.

Our study is based on direct student feedback on the curriculum and should be utilised to improve diagnostic teaching in early education. We would recommend revising existing medical curricula and continuing medical education programs to incorporate the concepts of overdiagnosis for both students and healthcare professionals. This ensures that future practitioners are educated to consistently consider the potential for harm alongside the potential benefits in their practice.

The understanding of high-value care is present in medicine however further insight is required to determine how students and doctors can maintain patient-centred care in clinical practice. Our model of learning pathways is useful for considering the potential impact of curriculum changes. Utilising the model in future research would allow for testing and refinement of the model.

The authors also recommend introducing clinical reasoning instruction as early as first year of medical school so students can gain a foundational understanding of the clinical reasoning process, which will prove invaluable as they encounter it firsthand during their clinical placements.

### Limitations

Our findings are limited to participants from one medical school, WSU, and data collection coincided with the COVID-19 pandemic. The interviews, however, were reflective and students described experiences prior to the pandemic and after the significant waves of the virus. Although the sample size of our study is numerically small, we were confident that we reached thematic saturation in this setting. Still, it would be valuable to conduct similar studies within other medical schools both nationally and internationally at different time periods for comparison.

The first author has limited qualitative research experience themselves, however, they have lived experience in the area. To enhance the credibility and integrity of the study the first author was supported by a highly experienced team. Dr Hegazi, the Director of Medical Education, enabled insight into the curriculum standards while researchers Dr Edmiston and Professor Peters shared critical insight and expertise regarding qualitative research standards and practices.

## Conclusion

Our study identified that throughout their medical school careers, medical students are exposed to overdiagnosis and low-value care and inherently begin to understand these concepts. There are differences, however, in the level of understanding between students that can be attributed to personal factors, the medical curriculum and clinical training setting. This study allows a unique perspective on how overdiagnosis currently features in medical education. Our findings can assist in targeting areas for improvement within medical education to promote a more patient-focused high-value care approach to clinical practice where students can begin to develop the foundations for this from their first day. Without a focus on diagnostic framework and strong clinical reasoning skills students will continue to struggle differentiating low and high-value care and will contribute to the problem of ‘too much medicine’.

### Electronic supplementary material

Below is the link to the electronic supplementary material.

Supplementary Material 1

## Data Availability

The datasets generated and analysed during the current study are not publicly available due to the requirements of the ethics approval but are available from the corresponding author on reasonable request.
