# Supplementary material for: Medical students’ awareness of overdiagnosis and implications for preventing overdiagnosis
Source: BMC Med Educ. 2024 Mar 8;24:256. doi: 10.1186/s12909-024-05219-2 (PMC10921797; doi:10.1186/s12909-024-05219-2)
Supplement: Supplementary file 1 — Supplementary Material 1 [file 12909_2024_5219_MOESM1_ESM.docx]

Supplementary File 1 – Interview Protocol

Interview Number: Date:

Good morning/afternoon, thank you for being here today to participate in this study. As stated in your consent form and information sheet, this interview will be audio recorded and transcribed, are you still happy to continue?

Thank you. For your privacy and confidentiality, I will refrain from using your name throughout the interview. If at any time you would like to stop the interview, please let me know. Since the interview is audio recorded only you are welcome to turn on your camera if you would like otherwise, we can get started with a few simple questions about yourself.

Age: Gender: Current Year Level:

Cultural background: Aboriginal and/or Torres Strait Islander:

International/Domestic Student (metro/rural): Current clinical school attachment:

**Clinical Scenario**

1. When seeing a new patient on the wards, if you are asked to assess them and make a diagnosis, what would you do?
   1. What was your most recent clinical attachment? Was there a patient that your team diagnosed? What was the process? What did you think about the process/outcome?

**Diagnosis**

1. Can you describe what you think the role of lab investigations and imaging is in diagnosis?
2. What do you think is the value in having a diagnosis?
   1. How do you decide what investigations to do after having identified differential diagnoses?
   2. How do you feel about not knowing exactly why the patient is experiencing symptoms?
3. How do you think patients feel about having a diagnosis?
4. Do you think a patient can be harmed by the process of diagnosis (or by receiving a diagnosis)?

**Diagnostic Teaching**

1. Reflecting on your preclinical years, can you describe how you learned about diagnosis?
   1. How do you feel PBL/ICM contributed to your diagnostic learning?
2. Now thinking about your clinical attachments, tell me what you have learnt about diagnosis.
3. Which rotation so far has taught you the most about making a diagnosis and why?
4. Have you ever asked for help or clarification with diagnosis? What response did you receive? How useful was the response and why?
5. Have you ever noticed missed opportunities for teaching about diagnosis while you are on the wards? What skills or topics do you feel are not taught enough?
6. What kinds of approaches to diagnosis have you observed while on clinical placement (on which rotations)?

**Overdiagnosis**

1. Have you heard of the term overdiagnosis? If so, where?
2. What is your understanding of the term overdiagnosis?
